# Supplementary material for: LSSmScarlet2 and LSSmScarlet3, Chemically Stable Genetically Encoded Red Fluorescent Proteins with a Large Stokes’ Shift
Source: Int J Mol Sci. 2022 Sep 21;23(19):11051. doi: 10.3390/ijms231911051 (PMC9569913; doi:10.3390/ijms231911051)
Supplement: Supplementary file 1 [file ijms-23-11051-s001.zip › ijms-1895632-supplementary.pdf]

Table S1. List of primers.

Primer	Primer sequence (5′-3′)	Comment
**mSc-BglII**	gacAGATCTATGGTGAGCAAGGGCGAG	Use for LSSmScarlet2 and LSSmScarlet3 and for mutagenesis of LSSmScarlet2
**mCherry-EcoRI-r**	tcgaattcttacttgtacagctcgtccatg	
**T74I-fw**	GTACGGCTCCAGGGCCTTCATCAAGCA CCCCGCCGATATC	Use for mutagenesis of LSSmScarlet2
**T74I-rv**	GATATCGGCGGGGTGCTTGATGAAGGC CCTGGAGCCGTAC	
**G170D-fw**	GATGGCCCTGCGCCTGAAGGACGGCGG CCGCTACCTGGC	Use for mutagenesis of LSSmScarlet2
**G170D-rv**	GCCAGGTAGCGGCCGCCGTCCTTCAGG CGCAGGGCCATC	
**H177D-fw**	GGCGGCCGCTACCTGGCAGACGTCAGG ACCACCTACAAG	Use for mutagenesis of LSSmScarlet2
**H177D-rv**	CTTGTAGGTGGTCCTGACGTCTGCCAG GTAGCGGCCGCC	
**L191Q-fw**	CAAGGCCAAGAAGCCCGTGCAGATGC CCGGCGCCTACAAC	Use for mutagenesis of LSSmScarlet2
**L191Q-rv**	GTTGTAGGCGCCGGGCATCTGCACGGG CTTCTTGGCCTTG	
**F217Y-fw**	CTACACCGTGGTGGAACAGTACGAACG CTCCGAGGGCCGC	Use for mutagenesis of LSSmScarlet2
**F217Y-rv**	GCGGCCCTCGGAGCGTTCGTACTGTTC CACCACGGTGTAG	
**EcoRI-D229G-rv**	CAGGAATTCTTACTTGTACAGCTCGTCC ATGCCGCCGGTGGAGTGGCGGCC	Use for mutagenesis of LSSmScarlet2
**LSSCy-KpnI**	tccggtaccgccaccATGGTGAGCAAGGGCGAG t	LSSmScarlet2 and LSSmScarlet3 into pAAV-CAG-R-GECO1-P2A-EGFP
**LSSCy-AgeI-r**	tgaaccggtcgCTTGTACAGCTCGTCCATG	LSSmScarlet2 and LSSmScarlet3 into pAAV-CAG-R-GECO1-P2A-EGFP
**NheI-LSSmSc**	tccgctagcggtcgccaccATGGTGAGCAAGGGCG AG	LSSmScarlet2 and LSSmScarlet3 into pTagBFP-actin at NheI/HindIII, pTagGFP2-tubulin at NheI/BsrGI sites
**LSSmSc-HindIII-r**	attaagcttGTACAGCTCGTCCATG	LSSmScarlet2 and LSSmScarlet3 into pTagBFP-actin at NheI/HindIII, pTagGFP2-tubulin at NheI/BsrGI sites
**mSc-BglII**	gacAGATCTATGGTGAGCAAGGGCGAG	LSSmScarlet2 and LSSmScarlet3 into pAAV-CAG-H2B-B-GECO1 at BglII/HindIII sites
**mCherry-HindIII-r**	GATAAGCTTTTACTTATACAGCTCGTC	LSSmScarlet2 and LSSmScarlet3 into pAAV-CAG-H2B-B-GECO1 at BglII/HindIII sites
**LSSmSc-XhoI**	agcctcgagATGGTGAGCAAGGGCGAG	LSSmScarlet2 and LSSmScarlet3 into pAAV-CAG-dMito-mCherry at XhoI/EcoRI sites (dMito = MSVLTPLLLRGLTGSARRLPVPRAKIHSLGPARSSVLTPLLLR GLTGSARRLPVPRAKIHSLGPARSAS)
**mCherry-EcoRI-r**	tcgaattcttacttgtacagctcgtccatg	LSSmScarlet2 and LSSmScarlet3 into pAAV-CAG-dMito-mCherry at XhoI/EcoRI sites (dMito = MSVLTPLLLRGLTGSARRLPVPRAKIHSLGPARSSVLTPLLLR GLTGSARRLPVPRAKIHSLGPARSAS)

**LSSmSc-BamHI**	ccgggatccaccggtcgccaccATGGTGAGCAAGGGCGAG	LSSmScarlet2 and LSSmScarlet3 into pLU-CMV-vimentin-NeonOxIrr at BamHI/BsrGI sites
**LSSmSc-XbaI-r**	tgatctagattaCTTGTACAGCTCGTCCATG	LSSmScarlet2 and LSSmScarlet3 into pLU-CMV-vimentin-NeonOxIrr at BamHI/BsrGI sites
**LA3-fw2**	GTTTTTGCTCTACGTTCTCCTGCTGGCCTTCTGCGCCTGTGCAGTGGGATTGATCGCCATTGGTGTAGC	LAMP3 into pAAV-CAG-dMito-LSSmScarlet at AgeI/BglII restriction sites
**LA3-r-BglII**	GTCAGATCTTAGATCCGCTTCGGCCTGCTTCAAGACAACCTGAACCGCTACACCAATGGCGATC	LAMP3 into pAAV-CAG-dMito-LSSmScarlet at AgeI/BglII restriction sites
**AgeI-LA3-fw1**	GACACCGGTGCCACCATGGCGGTGGAAGGAGGAATGAAGTGTGTCAAGTTTTTGCTCTACGTTCTC	LAMP3 into pAAV-CAG-dMito-LSSmScarlet at AgeI/BglII restriction sites
**Fw-BglII-TagBFP**	GCTCGAGATCTATGAGCGAGCTGATTAAGGAG	mTagBFP2 into pAAV-CAG-LAMP3-LSSmScarlet at BglII/EcoRI restriction sites
**Rv-EcoRI-TagBFP**	GCTCGGAATTCCTAATTAAGCTTGTGCCCCAGTTTG	mTagBFP2 into pAAV-CAG-LAMP3-LSSmScarlet at BglII/EcoRI restriction sites
**BglII-LSSmSc2**	CTAAGATCTGCTAGCCTCGAGATGGTGAGCAAGGGCGAG	LSSmScarlet3 into pAAV-CAG-LAMP3-mTagBFP2 at BglII/EcoRI restriction sites
**LSSCy-NheI**	ACCGCTAGCATGGTGAGCAAGGGCGAG	LSSmScarlet into pAAV-CAG-TagFTC-LAMP2A/R47H/T191A
**LSSCy-AgeI-r2**	TGAACCGGTCTTGTACAGCTCGTCCAT	LSSmScarlet into pAAV-CAG-TagFTC-LAMP2A/R47H/T191A
**hL1-r**	CTCCCACCGCTATGGGCACAAGGAAGTTGTCGTCATCTGCACTGCAGTCTTGGGCTGTAGAATACTTTCC	hLAMP2A into pAAV-CAG-LSSmScarlet-LAMP2A/R47H/T191A
**hL2-r**	GACCAATAAAATAAGCCAGCAACACTAGAATAAGTACTCCTGCCAAGGCAGCTCCCACCGCTATGGGCAC	hLAMP2A into pAAV-CAG-LSSmScarlet-LAMP2A/R47H/T191A
**AgeI-hLAMP2A**	AGAACCGGTGGCGGATCAATGGTGTGCTTCCGCCTC	hLAMP2A into pAAV-CAG-LSSmScarlet-LAMP2A/R47H/T191A
**hLAMP2A-XhoI-r**	TTCCTCGAGCTAAAATTGCTCATATCCAGCATGATGGTGCTTGAGACCAATAAAATAAGCCAG	hLAMP2A into pAAV-CAG-LSSmScarlet-LAMP2A/R47H/T191A
**BamHI-TagBFP2**	TGGGGATCCGCCACCATGAGCGAGCTGATTAAGGAG	mTagBFP2 into pAAV-CAG-LSSmScarlet-hLAMP2A
**TagBFP2-AgeI-r**	TGAACCGGTATTAAGCTTGTGCCCCAG	mTagBFP2 into pAAV-CAG-LSSmScarlet-hLAMP2A

**BamHI-LSSmSc2**	TGGGGATCCGCCACCATGGTGAGCAAGGGCGAG	LSSmScarlet2 and LSSmScarlet3 into pAAV- CAG-mTagBFP2-hLAMP2A
**LSSCy-AgeI-r2**	TGAACCGGTCTTGTACAGCTCGTCCAT	LSSmScarlet2 and LSSmScarlet3 into pAAV- CAG-mTagBFP2-hLAMP2A

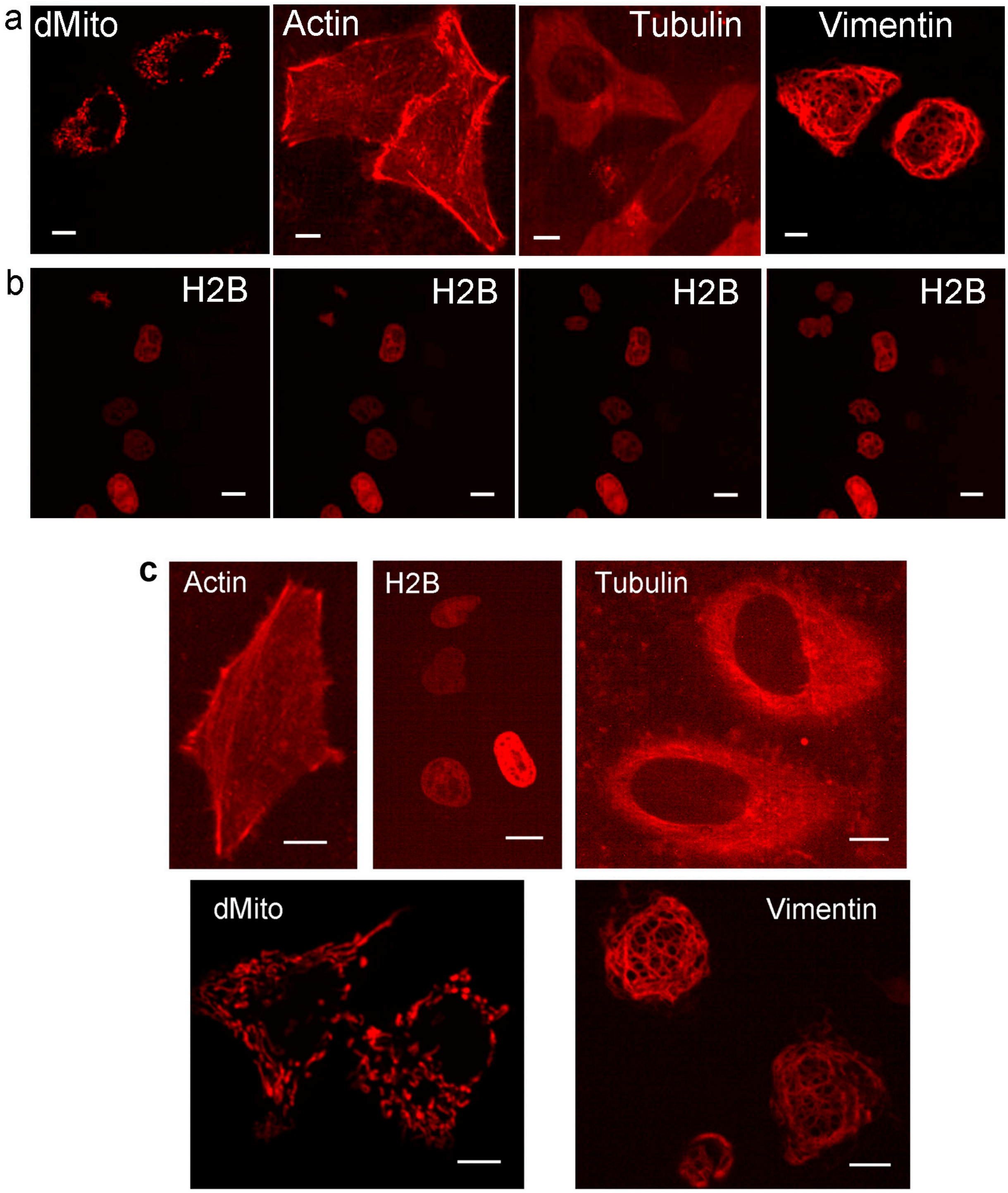

Figure S1. Localization of LSSmScarlet2 and LSSmScarlet3 proteins in different fusions in mammalian cells. (**a**) Confocal images of HeLa cells expressing the dMito-LSSmScarlet2, actin-LSSmScarlet2, tubulin-LSSmScarlet2 and vimentin-LSSmScarlet2 fusions. Scale bar, 50 μm. (**b**) Confocal images of non-dividing and dividing HeLa cells expressing the H2B-LSSmScarlet2 fusion. Scale bar, 50 μm. (**c**) Confocal images of HeLa cells expressing the actin-LSSmScarlet3, H2B-LSSmScarlet3, tubulin-LSSmScarlet3, dMito-LSSmScarlet3 and vimentin-LSSmScarlet3 fusions. Scale bar, 50 μm.

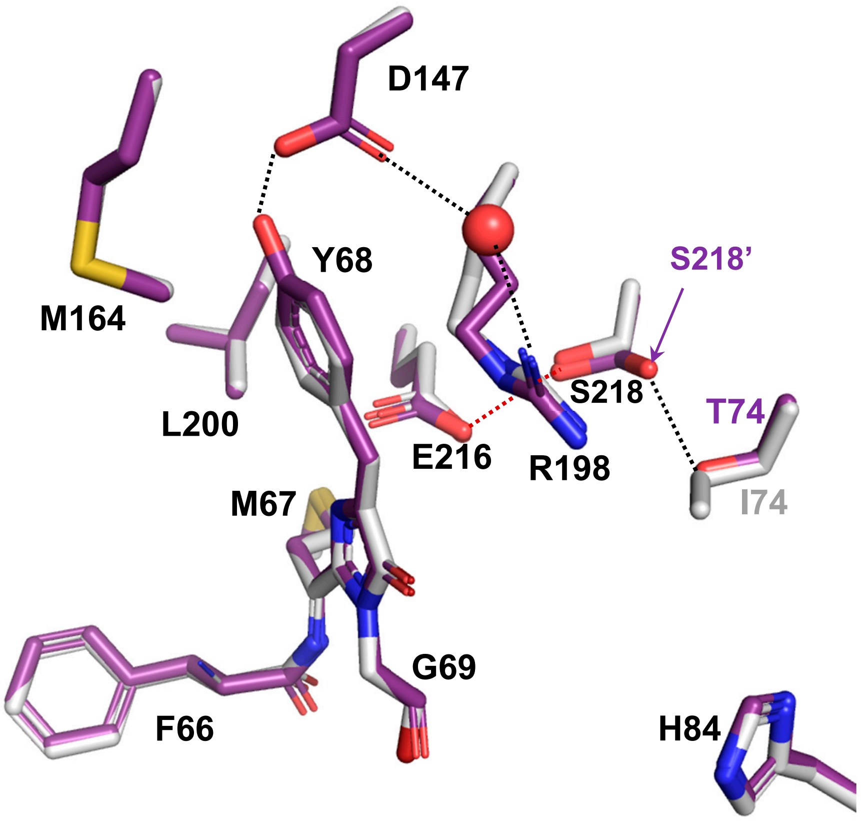

Figure S2. Comparison of the chromophore surrounding of LSSmScarlet2 (carbon atoms are colored in dark magenta) and LSSmScarlet (carbon atoms are colored in grey) .Label «S218'» indicates the alternative conformation of Ser218. Dashed lines indicate hydrogen bonds.

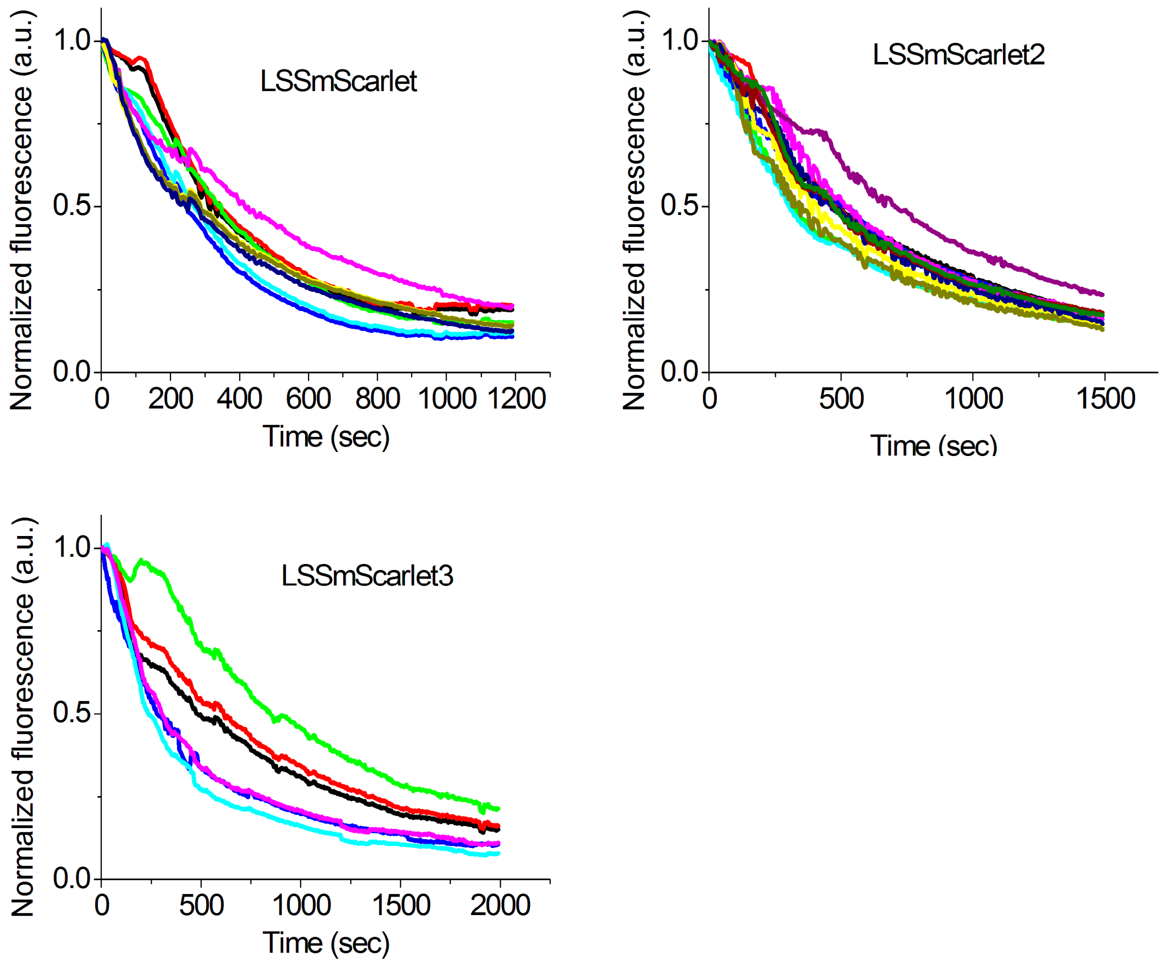

Figure S3. Raw photostability curves for the LSSmScarlet, LSSmScarlet2 and LSSmScarlet3 proteins.

Supplementary Methods.

Cloning of Bacterial Vectors, Mutagenesis and Library Screening

LSSRFP proteins were cloned into the pBAD/HisB plasmid (Invitrogen) at BglII/EcoRI restriction sites using the mSc-BglII/mCherry-EcoRI-r primers listed in Table S1 to express LSSRFPs proteins in BW25113 bacterial cells (kindly provided by Verkhusha V.V. from Albert Einstein College of Medicine, NY, USA).

Random libraries of LSSRFPs were obtained using PCR in the presence of Mn^{2+} ions (according to the Diversify PCR Random Mutagenesis Kit User Manual, Clontech, 2–3 random mutations were introduced per 1000 base pairs) and cloned at BglII/EcoRI restriction sites of the pBAD/HisB plasmid.

Screening of bacterial libraries was performed on Petri dishes under a fluorescent microscope. Briefly, expression of the LSSRFPs on the colonies on Petri dishes was induced with 0.02% arabinose for 24 h at 37 °C. Screening of about 10,000 colonies of the bacterial library expressing LSSRFPs variants was performed on Petri dishes under fluorescent stereomicroscope Leica M205FA (Leica, Germany) equipped with the DFC310FX camera (Leica Microsystems, Germany) and mercury metal halide light source EL6000 (Leica Microsystems, Germany). For screening on Petri dishes of pH-stable colonies we selected colonies with the highest fluorescence in the LSSRed channel (excitation at 480/40 nm and emission at 620/60 nm) in 1 h after spraying 100 mM NaOAc, pH 1.0. Acquired images were analyzed using ImageJ software, and colonies having the largest brightness were picked up for further analysis on bacterial lysates on Petri dishes. Next, using 96 well plate we analyzed the best 20-30 clones in *E.coli* bacterial lysates. We chose 1-2 clones based on the ratio of fluorescence at pH 7.5 to fluorescence at pH 3.5 that was more close to 1 for the best clones.

Mammalian Plasmids Construction

In order to construct the pAAV-*CAG*-LSSRFP-P2A-EGFP plasmids, the LSSRFP genes were PCR amplified as the KpnI-AgeI fragments, using LSSCy-KpnI/LSSCy-AgeI-r primers listed in the Table S1, and swapped with the R-GECO1 gene in the pAAV-*CAG*-R-GECO1-P2A-EGFP vector.

In order to construct the pLSSRFP-actin plasmid, the LSSRFP gene was PCR amplified as the NheI-HindIII fragment, using NheI-LSSmSc/LSSmSc-HindIII-r primers listed in Table S1, and swapped with the TagBFP gene in the pTagBFP-actin vector (Evrogen, Russia).

In order to construct the pLSSRFP-tubulin plasmid, the LSSRFP gene was PCR amplified as the NheI-BsrGI fragment, using NheI-LSSmSc/LSSmSc-HindIII-r primers listed in Table S1, and swapped with the TagGFP2 gene in the pTagGFP2-tubulin vector (Evrogen, Russia).

In order to construct the pAAV-CAG-H2B-LSSRFP plasmid, the LSSRFP gene was PCR amplified as the BglII-HindIII fragment, using mSc-BglII/mCherry-HindIII-r primers listed in Table S1, and swapped with the B-GECO1 gene in the pAAV-CAG-H2B-B-GECO1 vector [32].

In order to construct the pAAV-CAG-dMito-LSRFP plasmid, the LSSmRFP gene was PCR amplified as the XhoI-EcoRI fragment, using LSSmSc-XhoI/mCherry-EcoRI-r primers listed in Table S1, and swapped with the mCherry gene in the pAAV-CAG-dMito-mCherry vector.

In order to construct the pLU-CMV-vimentin-LSSRFP plasmid, the LSSmRFP gene was PCR amplified as the BamHI-BsrGI fragment, using LSSmSc-BamHI/LSSmSc-XbaI-r primers listed in the Table S1, and swapped with the NeonOxIrr gene in the pLU-CMV-vimentin-NeonOxIrr vector [33].

In order to construct the pAAV-CAG-LAMP3-LSSmScarlet plasmid, the LAMP3 gene was PCR synthetized as the AgeI-BglII fragment, using LA3-fw2/LA3-r-BglII and AgeI-LA3-fw1/LA3-r-BglII primers listed in the Table S1, and inserted in the pAAV-CAG-dMito-LSSmScarlet vector [9] at AgeI/BglII restriction sites.

In order to construct the pAAV-CAG-LAMP3-LSSmScarlet2 plasmid, the LAMP3 gene was PCR synthetized as the AgeI-BglII fragment, using LA3-fw2/LA3-r-BglII and AgeI-LA3-fw1/LA3-r-BglII primers listed in the Table S1, and inserted in the pAAV-CAG-dMito-LSSmScarlet2 vector described above at AgeI/BglII restriction sites.

In order to construct the pAAV-CAG-LAMP3-mTagBFP2 plasmid, the mTagBFP2 gene was PCR amplified as the BglII-EcoRI fragment, using Fw-BglII-TagBFP/Rv-EcoRI-TagBFP primers listed in the Table S1, and swapped with the LSSmScarlet gene in the pAAV-CAG-LAMP3-LSSmScarlet plasmid described above.

In order to construct the pAAV-CAG-LAMP3-LSSmScarlet3 plasmid, the LSSmScarlet3 gene was PCR amplified as the using BglII-LSSmSc2/mCherry-EcoRI-r primers listed in the Table S1, and swapped with the mTagBFP2 gene in the pAAV-CAG-LAMP3-mTagBFP2 plasmid described above.

In order to construct the pAAV-CAG-LSSmScarlet- LAMP2A/R47H/T191A plasmid, the LSSmScarlet gene was PCR amplified as the NheI-AgeI fragment using LSSCy-NheI/LSSCy-AgeI-r2 primers primers listed in the Table S1, and inserted in the pAAV-CAG-TagFTC-LAMP2A/R47H/T191A vector at NheI/AgeI restriction sites.

In order to construct the pAAV-CAG-LSSmScarlet-hLAMP2A plasmid, the hLAMP2A gene was PCR amplified from the pBoBi-hLAMP2-C-GC6s plasmid purchased from Addgene (#154151) as the AgeI-XhoI fragment using AgeI-hLAMP2A/hL1-r, AgeI-hLAMP2A/hL2-r and AgeI-hLAMP2A/hLAMP2A-XhoI-r primers primers listed in the Table S1, and inserted in the pAAV-CAG-LSSmScarlet- LAMP2A/R47H/T191A vector at AgeI/XhoI restriction sites.

In order to construct the pAAV-CAG-mTagBFP2-hLAMP2a plasmid, the mTagBFP2 gene was PCR amplified as the BamHI-AgeI fragment using BamHI-TagBFP2/TagBFP2-AgeI-r primers listed in the Table S1, and inserted in the pAAV-CAG-LSSmScarlet-hLAMP2A vector at BamHI/AgeI restriction sites.

In order to construct the pAAV-CAG-LSSmScarlet2-hLAMP2A plasmid, the LSSmScarlet2 gene was PCR amplified as the BamHI-AgeI fragment using BamHI-LSSmSc2/LSSCy-AgeI-r2 primers listed in the Table S1, and inserted in the pAAV- CAG-mTagBFP2-hLAMP2A vector at BamHI/AgeI restriction sites.

In order to construct the pAAV-CAG-LSSmScarlet3-hLAMP2A plasmid, the LSSmScarlet3 gene was PCR amplified as the BamHI-AgeI fragment using BamHI-LSSmSc2/LSSCy-AgeI-r2 primers listed in the Table S1, and inserted in the pAAV- CAG-mTagBFP2-hLAMP2A vector at BamHI/AgeI restriction sites.

Fixation of the Cells with 4% PFA and 2.5% GA.

Before fixation, cells in a 24 well plate with glass-bottom were washed with 1 ml of DPBS buffer. Next, fixation of the cells was performed with 1 ml of 4% PFA or 1 ml of 2.5% GA for 15 min at room temperature. The sample was further washed with 1 mL of DPBS buffer and kept in 1 ml of DPBS buffer for further imaging.

Supplementary References

9. Subach, O.M.; Vlaskina, A.V.; Agapova, Y.K.; Dorovatovskii, P.V.; Nikolaeva, A.Y.; Ivashkina, O.I.; Popov, V.O.; Piatkevich, K.D.; Khrenova, M.G.; Smirnova, T.A.; et al. LSSmScarlet, dCyRFP2s, dCyOFP2s and CRISPRed2s, Genetically Encoded Red Fluorescent Proteins with a Large Stokes Shift. *Int. J. Mol. Sci.* **2021**, *22*, 12887.

32. Subach, O.M.; Barykina, N.V.; Anokhin, K.V.; Piatkevich, K.D.; Subach, F.V. Near-Infrared Genetically Encoded Positive Calcium Indicator Based on GAF-FP Bacterial Phytochrome. *Int. J. Mol. Sci.* **2019**, *20*, 3488.

33 Subach, O.M.; Kunitsyna, T.A.; Mineyeva, O.A.; Lazutkin, A.A.; Bezryadnov, D.V.; Barykina, N.V.; Piatkevich, K.D.; Ermakova, Y.G.; Bilan, D.S.; Belousov, V.V.; et al. Slowly Reducible Genetically Encoded Green Fluorescent Indicator for In Vivo and Ex Vivo Visualization of Hydrogen Peroxide. *Int. J. Mol. Sci.* **2019**, *20*, 3138.
